# Dry care versus chlorhexidine cord care for prevention of omphalitis. Systematic review with meta-analysis

**DOI:** 10.1590/1518-8345.2695.3106

**Published:** 2019-01-31

**Authors:** María Dolores López-Medina, Manuel Linares-Abad, Ana Belén López-Araque, Isabel María López-Medina

**Affiliations:** 1 Universidad de Jaén, Jaén, Andalucía, Spain.; 2 Complejo Hospitalario de Jaén, Andalucía, Spain.

**Keywords:** Umbilical Cord, Chlorhexidine, Skin Care, Infection, Meta-Analysis, Infant, Newborn, Cordão Umbilical, Clorexidina, Higiene da Pele, Infecção, Metanálise, Recém-Nascido, Cordón Umbilical, Clorhexidina, Cuidados de la Piel, Infección, Metaanálisis, Recién Nacido

## Abstract

**Objective:**

to compare the effect of dry care and the application of chlorhexidine to the umbilical cord of newborns at risk of developing omphalitis.

**Method:**

systematic review with meta-analysis. Clinical trials comparing dry care with the application of clorexidine to evaluate omphalitis were selected. Methodological quality was evaluated using the *Consolidated Standards of Reporting Trials*.

**Results:**

the joint analysis of the studies shows a significant decrease in the risk of omphalitis in the chlorhexidine group compared to the dry care group (RR=0.58, CI: 0.53-0.64). However, in the analysis by subgroups, chlorhexidine umbilical cord care did not reduce the risk of omphalitis in hospital births (RR=0.82, CI: 0.64-1.05), in countries with a low infant mortality rate (RR=0.8, CI: 0.5-1.28), or at chlorhexidine concentrations below 4% (RR=0.55, CI: 0.31-1). Chlorhexidine acted as a protective factor at a concentration of 4% (RR=0.58, CI: 0.53-0.64), when applied in cases of home births (RR=0.57, CI: 0.51-0.62), in countries with a high infant mortality rate (RR=0.57, CI: 0.52-0.63).

**Conclusion:**

dry cord care is effective in countries with low infant mortality rate and in hospital births. However, 4% chlorhexidine for umbilical cord care protects against omphalitis in home births, in countries with a high infant mortality rate.

## RESUMO

**Objetivo:**

comparar o efeito da cura a seco e da aplicação de clorexidina no cordão umbilical de recém-nascidos em risco de desenvolver onfalite.

**Método:**

revisão sistemática com metanálise. Foram selecionados os ensaios clínicos que comparavam a cura a seco com a aplicação de clorexidina para avaliar a onfalite. A qualidade metodológica foi avaliada com *Consolidated Standards of Reporting Trials*.

**Resultados:**

a análise conjunta dos estudos mostra uma redução significativa do risco de onfalite no grupo da clorexidina em comparação com a cura a seco (RR=0,58; IC 0,53-0,64). Entretanto, na análise por subgrupos, a cura com clorexidina não reduziu o risco de onfalite em nascimentos hospitalares (RR=0,82; IC: 0,64-1,05), nos países com baixa taxa de mortalidade infantil (RR=0,8; IC: 0,5-1,28), ou com concentrações de clorexidina abaixo de 4% (RR=0,55; IC: 0,31-1). A clorexidina atuou como fator de proteção na concentração de 4% (RR=0,58; IC: 0,53-0,64), aplicada em nascimentos no domicílio (RR=0,57; IC: 0,51-0,62), em países com taxas de mortalidade infantil elevadas (RR=0,57; IC: 0,52-0,63).

**Conclusão:**

a cura a seco é eficaz em países com baixa taxa de mortalidade infantil e em nascimentos no contexto hospitalar. No entanto, a cura com clorexidina 4% protege contra a onfalite nos nascimentos domiciliares, em países com elevada mortalidade infantil.

## RESUMEN

**Objetivo:**

comparar el efecto de la cura seca y de la aplicación de clorhexidina en el cordón umbilical de los recién nacidos en el riesgo de desarrollo de onfalitis.

**Método:**

revisión sistemática con metaanálisis. Se seleccionaron ensayos clínicos que compararan la cura seca con la aplicación de clorhexidina evaluando la onfalitis. Calidad metodológica evaluada con *Consolidated Standards of Reporting Trials*.

**Resultados:**

el análisis conjunto de los estudios muestra una reducción significativa del riesgo de onfalitis en el grupo de clorhexidina en comparación con cura seca (RR=0,58; IC: 0,53-0,64). Sin embargo, en el análisis por subgrupos, la cura con clorhexidina no aportó reducción del riesgo de onfalitis en nacimientos hospitalarios (RR=0,82; IC: 0,64-1,05) en países con baja tasa de mortalidad infantil (RR=0,8; IC: 0,5-1,28), ni a concentraciones de clorhexidina inferiores al 4% (RR=0,55; IC: 0,31-1). La clorhexidina actuó como factor protector a concentraciones del 4% (RR=0,58; IC: 0,53-0,64), aplicada en nacimientos en el hogar (RR=0,57; IC: 0,51-0,62), en países con elevada mortalidad infantil (RR=0,57; IC: 0,52-0,63).

**Conclusión:**

la cura seca es eficaz en países con baja tasa de mortalidad infantil y nacimientos en ámbito hospitalario. Sin embargo, la cura con clorhexidina al 4% protege de onfalitis en nacimientos en el hogar, en países con elevada mortalidad infantil.

## Introduction

Omphalitis is an important cause of neonatal mortality and its prevention is of great importance for public health^1^. The incidence of omphalitis in newborns (NB) in developed countries is 0.7%, rising to 2.7% in developing countries^1^ ^-^ ^2^, and it affects both sexes equally^1^.

It is defined as a periumbilical acute bacterial infection with induration, erythema, bad smell, pain, and presenting or not association with purulent exudate at the base of the navel^3^. It is peculiar at the neonatal period, and the average age for its incidence is the third or fourth day of life^2^ ^-^ ^3^. 

The strategies for prevention of omphalitis are: hygiene practices at delivery, aseptic material to cut the umbilical cord and hand washing every time the cord is handled^4^. In the 21st century there have been several studies on umbilical cord (UC) care comparing different antiseptics, and several studies have shown that the hygiene habits of bathing and drying it were not associated with an increased risk of omphalitis when compared to alcohol application^4^ ^-^ ^6^. Topical triple dye is a treatment used in the United States, and there are several studies comparing the topical triple dye with alcohol application for UC care, and the results of these studies show that there are no differences between the treatment groups of omphalitis(^7-8)^. 

There are no studies with adequate level of evidence to establish recommendations on the most effective UC care for prevention of omphalitis in NB. Thus, a systematic review was performed to answer the question: Is the application of chlorhexidine more effective than dry cord care for prevention of omphalitis? The objective was: to compare the effect of dry care and the application of chlorhexidine to the umbilical cord of newborns at risk of developing omphalitis.

## Method

A systematic review with meta-analysis was carried out, for which a bibliographic search was performed in the Cochrane, Pubmed, Scopus, CINAHL, EMBASE, *Cuiden* and Spanish Medical Index (EMI) databases, and a reverse search with secondary recovery. The bibliographic search was carried out by January 2017, with no previous date range limit or language restriction. In order to identify the articles describing the incidence of omphalitis in NB to which dry care or chlorhexidine cord care was used for UC care, the following descriptors were used: *umbilical cord care*, *dry care*, *newborn*, *topical umbilical cord care*, *chlorhexidine umbilical cord care*, *umbilical cord care practices*, *randomized controlled trial* and *Clinical Trial*. The following search strategy was used in the PubMed/MEDLINE database: (Umbilical cord[mh] or cords, umbilical[tiab] or umbilical cord[tiab]) and (cord care[tiab] or dry care[tiab] or dry*[tiab] or chlorhexidine[mh] or chlorhexidine cord care[tiab]) and (new-born[mh] or infant[mh]) and (omphalitis[tiab]) and (clinical trial[pt]). To plan, prepare and publish the systematic review and meta-analysis, the guidelines provided by the Preferred Reporting Items for Systematic Reviews and Meta-Analyses (PRISMA)^9^ ^)^ were followed.

For the selection of the studies, two authors independently assessed the inclusion of the studies identified by the search strategy. In the first phase, the articles were selected according to their title and, after reading the abstracts, those that met the inclusion criteria were selected. Subsequently, an in-depth reading was carried out and their methodological quality was assessed using the Consolidated Standards Of Reporting Trials (CONSORT)^10^.

Clinical trials comparing dry care with the application of chlorhexidine solutions at all concentrations available for UC care were used as inclusion criteria. All living NB were included, without restricting the weight at birth, sex, gestational age, geographical area, level of development and delivery setting. 

Using a previously developed form, two authors independently extracted the data according to: type of study, population included, length of fieldwork period, duration of follow-up, type of intervention, procedure carried out with both the dry care and the chlorhexidine cord care, and results obtained. Those authors whose articles are the subject of this study were contacted so that they could provide the data necessary for performing the meta-analysis by subgroups. A third person evaluated the discrepancies found in order to decide on the inclusion of some articles and on data extraction.

The Grading of Recommendations Assessment, Development and Evaluation (GRADE)^11^ was used to evaluate the quality of the evidence, which was classified as: high, moderate, low or very low.

The results were expressed as relative risk (RR) with 95% confidence interval. The clinical heterogeneity and the homogeneity of the population were evaluated. The statistical heterogeneity and the consistency between the results of the studies were evaluated using I^2^ as criterion measure. I^2^ values of 25%, 50% and 75% were used to define heterogeneity as low, moderate and high. When this criterion was higher than 50%, a random effects model was applied to combine the results^12^. A sensitivity analysis of the results was carried out by performing several meta-analyzes sequentially, by subdividing them according to the methodological quality of the studies, the sample number and the concentration of chlorhexidine.

A subgroup analysis was performed for the data of the studies conducted with hospital and community NB, and dividing these data by the neonatal mortality rate (NMR) of the place of origin of the study: high NMR ≥10 per 1,000 live births versus low NMR <10 per 1,000 live births. In addition, a subgroup analysis was carried out for chlorhexidine concentrations: 4% chlorhexidine and chlorhexidine concentrations lower than 4%.

For the statistical analysis, Review Manager 5.3^13^ and Epidat 3.1^14^ softwares were used.

## Results

**Figure 1:**
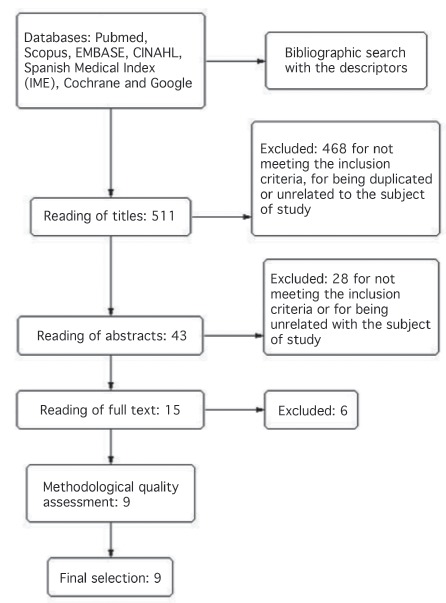
Process of selecting studies

Figure 1 shows the process of selecting studies. The literature search found 511 articles, of which 468 were discarded after a reading of their titles. The analysis of the summaries led to exclusion of 28 through a complete reading of 15 articles, and 6 were eliminated for different reasons: chlorhexidine was not compared with dry cord care^15^; be a research project^16^; not be a clinical trial(17); exclusively measure the time until the umbilical cord stump falls off^18^ ^-^ ^20^.

**Figure 2:**
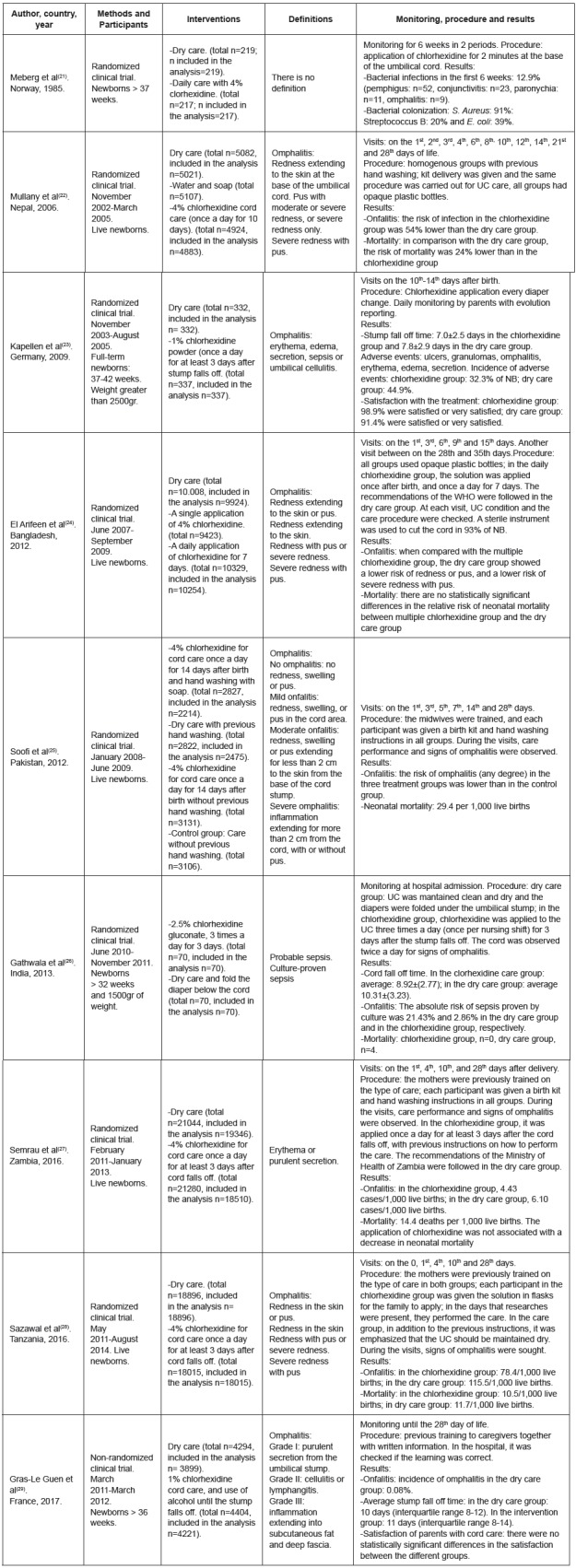
Characteristics of the studies included in the meta-analysis. Jaén, Andalusia, Spain, 2017

Figure 2 shows the characteristics of the sample of each study, interventions and outcome measures. 

The meta-analysis was performed using the 9 selected studies and 118,903 NB in total, of which 50.61% underwent dry umbilical cord care (60,182 NB). In total, there were 1,863 cases of omphalitis in both groups, and 64.03% of these cases of omphalitis belong to the dry cord care group.

**Figure 3:**
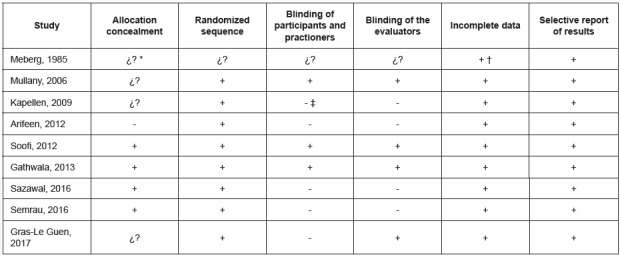
Biases of the studies included in the meta-analysis. Jaén, Andalusia, Spain, 2017

Figure 3 shows the biases of the different studies included in the meta-analysis, with no study considered to be invalid. 

* ¿? = Unknown risk, † + = Low risk of bias, ‡ - = High risk of bias

**Figure 4:**
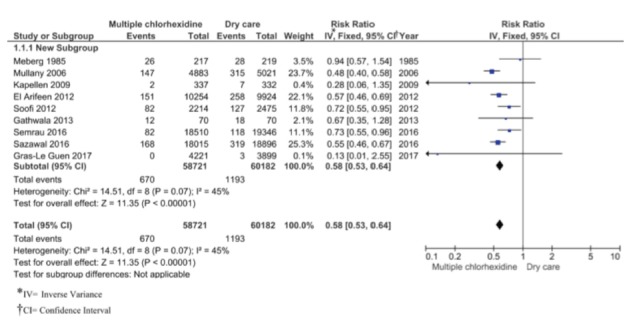
Omphalitis: Chlorhexidine vs Dry care

Regarding the risk of omphalitis, the 9 included studies show a significant decrease in the risk of omphalitis in the chlorhexidine group compared to the dry cord care group, with a RR of 0.58 (CI: 0.53-0.64), with moderate heterogeneity (I^2^=45%, χ^2^=14.51, p=0.07). This may be due to the clinical heterogeneity and, therefore, a subgroup analysis was performed. The result of the Egger’s test^30^ ^)^ was 0.4556 (p=0.6625), which indicates that there is no publication bias. The data with which the meta-analysis was carried out (Figure 4) come from studies in which chlorhexidine was applied multiple times. According to the GRADE system, this level of evidence is rated as moderate. It can be seen in the tree graph (Figure 4) that four studies^21^ ^,^ ^27^ ^-^ ^29^ do not show a significant decrease in the risk of omphalitis with the use of chlorhexidine for cord care when compared with dry care.

**Figure 5:**
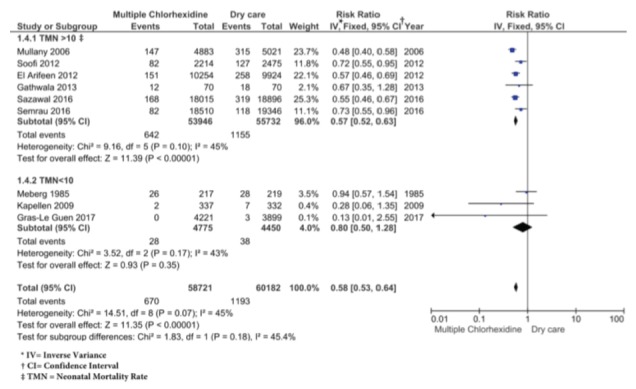
Omphalitis: Chlorhexidine vs Dry care, according to the Neonatal Mortality Rate

When a subgroup analysis was performed, it is observed that in countries with NMR<10 there was a RR of 0.80 (CI: 0.5-1.28), so there are no significant differences between the two types of care for prevention of omphalitis. However, there is a significant decrease in the risk of omphalitis in the subgroup with NMR>10 (RR=0.57, CI: 0.52-0.63), as show in Figure 5. Countries with NMR<10 are those in which the studies are conducted with NB older than 36 weeks. According to the GRADE system, the level of evidence is moderate for those studies with NMR>10 and low for the present research with NMR<10.

In the community births group there is a significant decrease in the risk of omphalitis using chlorhexidine for umbilical cord care (RR=0.57, CI: 0.51-0.62), and a moderate heterogeneity of data was obtained (I^2^=45%, χ^2^=7.3, p=0.12). In the community births group there is a significant decrease in the risk of omphalitis using chlorhexidine for UC care (RR=0.57, CI: 0.51-0.62), and a moderate heterogeneity of data was obtained (I^2^=45%, χ^2^=7.3, p=0.12). These data from the meta-analyzes by subgroups correspond to a level of evidence rated as moderate, according to the GRADE system.

The sensitivity analysis shows that the risk of omphalitis remains the same for all studies by excluding the studies in which blinding is not performed (RR=0.54, CI: 0.47-0.61). When studies not presenting selection bias are analyzed, the risk increases, but there is still a significant decrease in the risk of omphalitis with the use of chlorhexidine for UC care (RR=0.63, CI: 0.55-0.72), and in this case, the statistical heterogeneity obtained is low (I^2^=23%). When sensitivity was analyzed by eliminating studies, it is observed that if a study is eliminated^22^, the resulting relative change is 6.18%, and this is the research whose confidence interval is more distant from 1. 

Regarding the different concentrations of chlorhexidine used in the studies, when the meta-analysis is performed only with the studies in which a 4% chlorhexidine concentration was used in the intervention group, the result is a RR=0.58; CI: 0.53-0.64. By jointly analyzing the studies in which concentrations lower than 4% were used, a RR=0.55 is obtained; CI: 0.31-1. The high heterogeneity of the studies prevents an independent analysis for chlorhexidine concentrations of 1% and 2.5%.

## Discussion

In a joint analysis, with the inclusion of the latest published studies, the current evidence showed a significant decrease in the risk of omphalitis with the use of multiple applications of chlorhexidine when compared with dry cord care. In countries with high neonatal mortality rates, such as Nepal, with 22 deaths per 1,000 live births^31^, the risk of omphalitis is lower with the application of chlorhexidine when compared with dry cord care. In contrast, in countries with very low neonatal mortality rates, such as Germany, with 2 deaths per 1,000 live births^31^, application of chlorhexidine does not differ from dry care in relation to the risk of omphalitis, although these studies investigated a small sample when compared with those whose NMR>10. 

The results also show that community births present a lower risk of omphalitis with the application of chlorhexidine, a finding that corroborates the data from a review conducted in 2015, in which RR=0.48; CI: 0.4-0.57^32^, and from another one carried out in 2016, in which RR=0.4; CI: 0.25-0.63, with an I^2^ of 68%^33^. This situation is not consistent with the findings of studies with a group of hospital births, in which there were no differences between the application of chlorhexidine and dry care. The findings of other studies also do not show differences in the incidence of omphalitis depending on the type of cord care, although they did not compare solely dry care with the application of chlorhexidine^32^ ^-^ ^33^ for UC care.

Several systematic reviews have shown similar results, in which chlorhexidine was found to reduce the risk of omphalitis^33^ ^-^ ^35^, especially in countries with high NMR. In this sense, our results support that UC care with the use of 4% chlorhexidine protects against omphalitis in home births in countries with high NMR. The application of chlorhexidine in concentrations lower than 4% did not act as a protection factor against omphalitis, although it must be emphasized that the studies using these concentrations of chlorhexidine evaluated hospital births. 

Depending on where the birth takes place, the UC cutting technique is performed with the use of a new or boiled razor blade^35^ ^-^ ^36^, and this, together with the lack of hand washing before the intervention^35^ increases the risk of infection, especially in home deliveries. Researchers are aware that efforts to promote hand washing, cut the umbilical cord with the use of clean instruments and avoid unclean domestic interventions can reduce exposure to infectious agents and improve neonatal outcomes^37^.

Limitations: This systematic review with meta-analysis needs to be interpreted with caution due to the included clinical trials and their own limitations. In at least 5 of these studies, it was not possible to mask the intervention of participants and professionals, although it is unlikely that the results were biased, as proven by the sensitivity analysis.

There is variation in the interventions carried out in the different studies such as: in 4 research studies^22^ ^,^ ^25^ ^,^ ^27^ ^-^ ^28^, training was provided to the mothers so that they could perform a correct hand hygiene. Regarding hygiene for cutting of the UC, 5 studies^22^ ^,^ ^24^ ^-^ ^25^ ^,^ ^27^ ^-^ ^28^ specify that a delivery kit was given to achieving maximum cleanliness.

There were 3 studies^22^ ^,^ ^24^ ^,^ ^25^ in which the delivery took place in community settings, 2 in community and hospital settings^27^ ^-^ ^28^ and four in hospital settings^21^ ^,^ ^23^ ^,^ ^26^ ^,^ ^29^.

In 6 studies, the chlorhexidine concentration used for cord care was 4%^21^ ^-^ ^22^ ^,^ ^24^ ^-^ ^25^ ^,^ ^27^ ^-^ ^28^, and the concentrations used in the remaining three studies^23^ ^,^ ^26^ ^,^ ^29^ were 2.5% and 1%^23^ ^,^ ^26^ ^,^ ^29^. The sensitivity analysis performed considering the different chlorhexidine concentrations used, suggests that the use of chlorhexidine in concentrations lower than 4% is not associated with a greater protection against omphalitis than that provided by dry umbilical cord care.

Another limitation of the present analysis is that no data on low birth weight and premature babies are shown. The analysis only used data available from studies whose inclusion criteria specified NB at more than 36 weeks gestation.

The criteria used to perform the analysis on the studies classifying omphalitis into several categories were: Redness with pus or severe redness and severe redness with pus, which correspond to moderate and severe omphalitis.

There is no conflict of interest or funding in this study.

## Conclusion

Application of 4% chlorhexidine in NB significantly decreases the incidence of omphalitis in home births in countries with a NMR higher than 10 deaths per 1,000 live births. The inclusion of newly published studies reinforces the level of evidence so that the use of chlorhexidine is recommended for UC care in developing countries. This meta-analysis provides important information for the policies aiming at the care for NB in home births and in high-risk situations where hygiene conditions are not appropriated. 

There are no significant differences between dry cord care and the use of chlorhexidine in concentrations lower than 4% for UC care in countries with low NMR and in hospital births. It was evidenced that dry cord care is an effective intervention in these contexts and it may be recommended for prevention of omphalitis because it is less expensive. Therefore, it is convenient to expand the knowledge through double blind clinical trials in these contexts to evaluate both interventions and thus improve the care practice provided to the newborn.

In full term NB, there are no statistically significant differences between the two groups of UC care. It is necessary to carry out more studies according to the gestational age to know what proportion of preterm newborns have omphalitis regardless of the type of cord care. 

It would be useful to conduct studies with qualitative methodology to know the experiences in the UC care and consider them for the development of more effective and efficient health strategies aiming at reducing the incidence of omphalitis.
